# Tuning photosynthetic oxygen for hydrogen evolution in synergistically integrated, sulfur deprived consortia of *Coccomyxa chodatii* and *Rhodobium gokarnense* at dim and high light

**DOI:** 10.1007/s11120-022-00961-4

**Published:** 2022-11-23

**Authors:** Amal W. Danial, R. Abdel-Basset, Huwida A. A. Abdel-Kader

**Affiliations:** Botany and Microbiology Department, Faculty of Science, Assiut University, Assiut, Egypt

**Keywords:** *Coccomyxa chodatii*, *Rhodobium gokarnense*, PNSB, Hydrogen evolution, Sulfur deprivation, Photosynthesis

## Abstract

In this work, tuning oxygen tension was targeted to improve hydrogen evolution. To achieve such target, various consortia of the chlorophyte *Coccomyxa chodatii* with a newly isolated photosynthetic purple non-sulfur bacterium (PNSB) strain *Rhodobium gokarnense* were set up, sulfur replete/deprived, malate/acetate fed, bicarbonate/sulfur added at dim/high light. *C. chodatii* and *R. gokarnense* are newly introduced to biohydrogen studies for the first time. Dim light was applied to avoid the inhibitory drawbacks of photosynthetic oxygen evolution, values of hydrogen are comparable with high light or even more and thus economically feasible to eliminate the costs of artificial illumination. Particularly, the consortium of 2*n*− (*n* = 1.9 × 10^5^ cell/ml, sulfur deprived) demonstrated its perfection for the target, i.e., the highest possible cumulative hydrogen. This consortium exhibited negative photosynthesis, i.e., oxygen uptake in the light. Most hydrogen in consortia is from bacterial origin, although algae evolved much more hydrogen than bacteria on per cell basis, but for only one day (the second 24 h), as kinetics revealed. The higher hydrogen in unibacterial culture or consortia results from higher bacterial cell density (20 times). Consortia evolved more hydrogen than their respective separate cultures, further enhanced when bicarbonate and sulfur were supplemented at higher light. The share of algae relatively increased as bicarbonate or sulfur were added at higher light intensity, i.e., PSII activity partially recovered, resulting in a transient autotrophic hydrogen evolution. The addition of acetic acid in mixture with malic acid significantly enhanced the cumulative hydrogen levels, mostly decreased cellular ascorbic acid indicating less oxidative stress and relief of PSII, relative to malic acid alone. Starch, however, decreased, indicating the specificity of acetic acid. Exudates (reducing sugars, amino acids, and soluble proteins) were detected, indicating mutual utilization. Yet, hydrogen evolution is limited; tuning PSII activity remains a target for sustainable hydrogen production.

**Supplementary Information:**

The online version contains supplementary material available at 10.1007/s11120-022-00961-4.

## Introduction

Algae, bacteria, and cyanobacteria are repeatedly studied to catalyze hydrogen evolution via their photosynthetic or fermentative pathways. Bacteria are frequently used to hydrolyze biomass and produce biohydrogen, photosynthetic PNSB (Danial et al. 2017; Abdel-Kader et al. 2022) or heterotrophic (Danial et al. 2015; Danial and Abdel-Basset 2015; Gad El-Rab et al. 2018**).** Algae-bacteria consortia, through various aspects linking their metabolism, may yield efficiently enhanced hydrogen production. Bacteria and algae can proceed integrative metabolism of photosynthesis and respiration (oxygen and carbon dioxide exchange), light spectra absorption and utilization of secreted metabolites, particularly acetic acid (Fakhimi et al. 2020). To induce hydrogen evolution swiftly, sulfur deprivation was developed and established in *Chlamydomonas reinhardtii* since Melis et al. (2000). This two-stage hydrogen evolution, at which oxygen evolution ceases along with starch accumulation/fermentation at a closed system, thus creating anaerobic conditions for hydrogen evolution. Such induction, however, is transient and typically lasts for up to a number of (3–4) days**.** A characteristic effect of sulfur limitation is the decline in the expression of many photosynthetic genes, encoding subunits of photosystem I (PSI), photosystem II (PSII) and ATPase, whereas the transcript levels of two particular antenna proteins, LHCBM9 and LHCSR1 are upregulated (Nguyen et al. 2011; Toepel et al. 2013). Response in gene expression occurs already after a few hours of sulfur deprivation (González-Ballester et al. 2008); during which most photosynthetic genes, Rubisco and antenna protein genes are downregulated (Toepel et al. 2013). The inactivation of PSII is assumed to be caused by the strongly oxidizing species Tyrz^+^ and P680^+^ upon illumination, leading to donor-side-induced photoinhibition, resulting in the relatively rapid degradation of PSII reaction center proteins, including PsbA, PSBO, CP43 and possibly several more subunits (reviewed by Antal et al. 2015; Nagy et al. 2018a). Upon sulfur deprivation, the expression level of the nuclear-encoded PSBO subunit, which normally has a relatively long lifetime (Nelson et al. 2014), is also downregulated (Toepel et al. 2013), possibly contributing to the decrease in PSII activity. The rapid loss of CP43 was unexpected; in the absence of this protein, PSII is destabilized (Sugimoto and Takahashi, 2003), which may also contribute to the loss of photosynthetic activity. Besides, Nagy et al. (2016, 2018a) proposed that with the overexcitation of PSII, ^1^O_2_ singlet oxygen in PSII are produced exerting oxidative stress, which triggers ascorbate biosynthesis; when accumulated to the mM concentration range, ascorbate may contribute to the inactivation of the Mn-cluster in the OECs (oxygen evolving complexes) due to its reducing capacities and may provide electrons to PSII, albeit at a low rate.

Sulfur deprivation also leads to strong starch and phosphatidylglycerol accumulation (Sugimoto et al. 2007, 2008) and the alteration of the cell wall structure **(**Takahashi et al. 2001). On the other hand, respiration is maintained, which, together with the loss of PSII activity, leads to the establishment of hypoxia, essential for the expression and activity of the highly O_2_-sensitive hydrogenases and nitrogenases (Ghirardi et al. 1997; Abdel-Basset and Bader 1997, 2008; Abdel-Basset et al. 1998; Milligan et al. 2007) which catalyze hydrogen evolution in algae and bacteria, respectively. The purple anoxygenic photosynthetic bacterium *Rhodospirillum rubrum* displayed lack of growth, cessation of bacteriochlorophyll and protein accumulation and inhibition of H_2_ evolution although most cells remained viable after 100 h of S-deprivation (Melis and Melnicki 2006; Melnicki et al. 2009). All of its nitrogenase functions including N_2_-fixation are promptly downregulated at multiple levels in response to S-deprivation, preventing hydrogen production. Instead, cell volume increased, and large amounts of polymer poly-*b*-hydroxybutyrate (PHB) were found to accumulate extracellularly (3.5-fold within 24 h of S-deprivation). Similarly, *R. rubrum*, as *Rhodobacter sphaeroides and Rhodopseudomonas palustris* exhibited a similar response to S-deprivation (Melnicki et al. 2009).

Despite such consequences of sulfur deprivation, cell sulfur decreased by only 25% (Nagy et al. 2016) and, furthermore, sulfur reserves (membrane sulfolipids) mobilize upon sulfur deprivation (Sugimoto et al. 2007, 2008, 2010).

Light and bicarbonate orchestrate photosynthetic oxygen evolution and sulfur re-addition might recover PSII to resume its activity to evolve oxygen. Other than carbon fixation in Calvin–Benson (CB) cycle and carbon concentrating mechanism (CCM) (Hong et al. (2016), bicarbonate affects PSII activity via Warburg’s effect (Govindjee and coworkers, reviewed in Shevela et al. 2012). Light intensity and quality have been studied in the field of hydrogen evolution (e.g., Jurado-Oller et al. 2015). Light might turn stressful not only to photosynthesis and hydrogen evolution by inducing high rates of oxygen evolution that inhibits hydrogenases but also to microbial life itself by production of singlet oxygen species. Down regulation is among the adaptive mechanisms that protects of PSII from photooxidation induced by high light intensity (Abdel-Basset et al. 2011).

Therefore, this work was planned to enhance the hydrogen evolution capacity of the green alga *Coccomyxa chodatii* SAG 216-2 and a local, newly isolated photosynthetic purple non-sulfur bacterium (PNSB) (*Rhodobium gokarnense*), via tuning oxygen content, the powerful inhibitor of the hydrogen evolving enzymes hydrogenases and nitrogenases. To approach the relevant oxygen partial pressure, several manipulations were applied: namely, algae-bacteria consortia, sulfur deprivation, light intensity, and organic acid (malic and acetic) supplementation. A relevant oxygen partial pressure that guarantees the balance between photosynthetic electron flow for proton reduction on the one hand but not inhibitory to the enzymes on the other hand is a highly complicated network of metabolic processes. Whether cessation of hydrogen evolution is reversible, attributed to cells death or nutrient depletion are questions to be answered in this work via supplementing more nutrients (bicarbonate or sulfur). Organic acids (malic or malic/acetic mixture) are supposed to enhance respiration of both organisms, thus, anaerobiosis in addition to electron supplementation. A minimum (dim) light intensity has been applied to avoid overreduction of the electron transport components and their subsequent deterioration as well as eliminating the costs of artificial light if applicable.

## Materials and methods

### Algal culture and growth

Cultures of the green alga *C. chodatii* SAG 216-2 (kindly offered by SAG, Germany) were cultivated under sterile conditions in modified Bold Basal medium (BBM, Bischoff and Bold 1963) at a temperature of 25 ± 2 °C, pH of 6.8 ± 0.1, illuminated continuously with white, fluorescent lamps (50 μmol/m^2^/s) and agitated by orbital shaker at 150 rpm for 7 days. Cultures of *C. chodatii* were subjected to sulfur deprivation by replacing sulfate salts in BBM by their analogous chlorides and designated throughout the whole manuscript as C+ and C− (sulfur replete and sulfur deprived, respectively).

### Bacterial isolation, growth and characterization

Strains of photosynthetic PNSB were isolated from local ponds in Assiut University, Assiut, Egypt and grown anaerobically in RÄH medium (Biebl and Pfennig 1981) at 30 °C and a white light intensity of about 70 μmol/m^2^/s. After 1–2 weeks of incubation, a purplish red color developed in the medium and the PNSB colonies were characterized morphologically and biochemically in addition to molecular identification. They were analyzed macroscopically considering pigmentation, colony length and width; the colony size and shape were determined using light microscopy and the genotypic characterizations of the different isolates were assessed. The PNSB cultures “R” were designated throughout the whole manuscript as R+ and R− (sulfur replete and sulfur deprived, respectively).

### Phylogenetic analysis of bacteria

The morphological, biochemical and molecular characteristics were used for strain characterization, according to Bergey’s Manual of Systematic Bacteriology (Brenner et al. 2005): bacterial strains were identified based according to the partial 1500 bp sequences of 16Sr RNA of the strains, and comparison in the GenBank databases.

Total genomic DNA was extracted and purified from the samples. The primer set of F (5-AGA GTT TGA TCC TGG CTC AG-3) with a GC clamp and R (5-GGT TAC CTT GTT ACG ACT T-3) at the annealing temperature of 65 °C were used for the PCR amplification of the variable region of 16S rDNA from the purified genomic DNA. Then, we made PCR clean up to the PCR product using GeneJET™ PCR Purification Kit (Thermo). Loading to 4 µl from the PCR mixture was carried out to examine the PCR product on 1% agarose gel against 1 Kb plus ladder (Fermentas). Finally, sequencing to the PCR product on GATC Company using ABI 3730xl DNA sequencer, forward and reverse primers were conducted. Sequence analysis was achieved by searching through online databases using BLAST. The phylogenetic analysis was performed using MEGA 3.1 software. The phylogenetic tree was constructed by the Neighbor Joining method. The sequences obtained were compared with available database sequences using a BLAST search.

### Experimental set up for growth and hydrogen evolution

Various algal/bacterial consortia were set up by mixing different volumes of the green alga *C. chodatii* SAG 216-2 (C+/−) with the photosynthetic PNSB *R. gokarnense* (R+/−), either sulfur replete (+) or sulfur deprived (−). The consortia were subjected to sulfur deprivation by replacing sulfate salts by their analogous chlorides. These consortia are designated throughout the work as 1*n*+, 1*n*−, 2*n*+, 2*n*−, 3*n*+, 3*n*−, 4*n*+, 4*n*−; *n* indicates cell number of bacteria (1.8 × 10^5^ cells/ml) and algae (0.085 × 10^5^ cells/ml) giving together a final optical density of about 0.25 A at 0-time of cultivation. However, the consortium of 3*n* refers to or equals 2*n*(R) + *n*(C). The same O.D.750 nm was containing much less algal (1/20^th^) than bacterial cell numbers, due to the larger algal cell size. These consortia were followed for growth, hydrogen evolution and the related metabolic processes.

Bottles containing hydrogen evolving cocktails (10% phosphate buffer pH 6.8 ± 0.2, 10% a mixture of bacteria and algae at early log phase, 80% medium to obtain a total volume of 1 l) were stoppered and flushed with nitrogen for 15 min. They were then kept at a light intensity of 2 or 70 µmol/m^2^/s (tungsten lamp), 25 °C and shaken at 150 rpm as long as hydrogen was evolving. Two sets of experiments were identically set up, one for hydrogen collection and another set for tracing growth of algae and bacteria.

### Hydrogen collection and detection

The gas evolved from the different culture cocktails was passed over NaOH solution (700 ml 1 M) to absorb carbon dioxide and then collected by replacing water in a water-filled graduated cylinder inverted in water. Gas samples at the head space of the fermentation bottles were checked for hydrogen purity using FID-TCD gas chromatography (Thermo Scientific TRACE GC Ultra, Germany).

### Treatments

Algal and bacterial consortia were grown in BB medium that has been subjected to the following modifications and amendments:

#### Malic acid supplementation

Culture combinations, suspended in BBM, were enriched with malic acid at the same concentration of RÄH medium (Biebl and Pfennig 1981), as the basic medium to evolve hydrogen by the PNSB.

#### Malic and acetic acids supplementation

In addition to malic acid, acetic acid was added to BBM at 1 ml/l, the concentration conventionally supplemented in TAP medium for *Chlamydomonas reinhardii*.

#### Sulfur deprivation

Two sets of experiments, one sulfur replete (+S) and sulfur deprived (−S) of *Coccomyxa codtatii*, *R. gokarnense* and their consortia were studied at the above-described hydrogen evolution cocktails.

#### Bicarbonate addition

After cessation of hydrogen evolution (4 days), bicarbonate (sodium salt) at two concentrations of 5 and 10 mM were added to the cultures aiming to resume autotrophic metabolism and its impact on hydrogen evolution was recorded. Only the results of 10 mM bicarbonate are presented as they induced more hydrogen than 5 mM bicarbonate.

#### Sulfur re-addition

After cessation of hydrogen evolution induced by bicarbonate, sulfur (magnesium sulfate 0.30 mM), aiming to resume regular (sulfur replete) metabolism and its impact on hydrogen evolution.

#### Light intensity

The above-described experiments were performed at dim light of 2 µmol/m^2^/s (room light at winter) or higher light intensity of 70 µmol/m^2^/s (two tungsten lamps).

### Analytical methods

#### Optical density and cell number

Optical density at 750 nm (O.D.750 nm) of all consortia was followed daily over the growth period. Cell number of algae was counted using an Improved Neubauer ruled hemocytometer.

#### Bacterial cell number counting

Using the dilution plate method, bacterial colonies developed after growth for 24–48 h at RÄH agar medium, were counted as the colony forming units (CFU).

#### Photosynthesis and respiration

The net photosynthetic oxygen evolution (*P*_N_) and dark respiratory oxygen uptake (R_D_) were monitored daily using a Clark type electrode computerized to an Oxygen Monitoring System (OMS, Hansatech Instruments Inc., donation from the Alexander von Humboldt Foundation Germany to R. Abdel Basset). Two milliliters of algal cultures and consortia were followed under continuous red-light intensity of 2 and 70 μmol/m^2^/s (growth lights) at 25 °C for 15 min; the rate of P_N_ was calculated as nmole O_2_↑ µg^−1^ Chl/h. Respiration (*R*_D_) was also monitored as O_2_ uptake using the above system, but in the dark; the rate of *R*_D_ was calculated as nmole O_2_↓ µg^−1^ Chl/h.

### Estimation of metabolites

#### Chlorophylls and carotenoids assessment

Chlorophylls (a and b) and carotenoids contents were assessed in ethanol extracts according to Arvola (1981), calculated and expressed as µg/ml algal suspension according to Metzner et al. (1965).

#### Bacteriochlorophyll (B.Chl) assessment

Bacteriochlorophyll of the *R. gokarnense* were assessed substantially as in *R. cupsulata* following Sojka et al. (1970) in a series of dilutions of the bacterium intact cells based on that of Barer (1955) and Namsaraev (2009). Absorbances at 860, 660 and 775 nm were measured (in cuvettes with 1-cm light path). The A_860_–A_660_ differences were calculated and used to calculate B.Chl/ml of culture utilizing an extinction coefficient of 75 mM/cm (Clayton 1963); absorbances at 775 nm were treated the same way. Ordinary aqueous suspensions, ratios of peak-to-trough absorbances (e. g., 860 nm relative to 660 nm) are high (Sojka et al. 1970), suggesting that the light-scattering background might not contribute greatly to the total absorbance observed at the 860 nm. B.Chl peak. A correlation between cell number and bacteriochlorophyll was set up. Estimating bacteriochlorophyll in intact cells saves time, solvent costs, solvent hazards and protective from in vitro degradation.

#### Ascorbate assessment

Ascorbate assessment was performed substantially according Jagota and Dani (1982). Aliquots of 2 ml culture was sonicated for 2 min (Sonicator Bandelin Sonopuls HD 3200 homogenizer in Ultrasonic, Germany) in 5% TCA (trichloroacetic acid), then centrifuged at 3000 rpm for 5 min; the supernatant was used for ascorbate determination. Ascorbate contents were substantially assessed according Jagota and Dani (1982). A volume of 0.2 ml of the supernatant, 0.8 ml of 10% TCA, 1 ml H_2_O and 0.2 ml Folin (diluted) were mixed well and left for 10 min; the developed blue color was read at 760 nm using spectrophotometer.

#### Starch determination

At the end of the growth period, 2 ml of the cultures were boiled in glass tubes containing ml ethanol (80%) for 2 h.; after centrifugation, the supernatants were decanted. The collected pellets were resuspended in 5 ml of diluted perchloric acid solution (9.2 mM) for 30 min at 100 °C to dissolve starch. After centrifugation the supernatant was used for determining reducing sugars resulting from starch hydrolysis following the procedure of Miller (1959).

### Cell exudations

Batches of the green alga and the bacterium consortia were grown at conditions identical to those of hydrogen photoevolution i.e., for 7 days at light intensity of 2 μmol/m^2^/s, once at aerobic and another at anaerobic conditions. In unialgal, unibacterial and Co-culture exudates, reducing sugars, amino acids and soluble proteins were determined as exudates into the growth media (50 ml); separated from cells by centrifugation (2000×*g*). The reducing sugars were determined following the procedure of Miller (1959); amino acids according to the method of Moore and Stein (1948) and soluble protein contents using the method of Lowry et al. (1951), using egg albumin as the standard protein.

### Statistical analysis

Each experiment was repeated three times and the mean values of three replicates ± standard error (se) are presented. Statistical analysis of the data was conducted using ANOVA one-way test (analysis of variance) by SPSS program version 21, and Duncan values were determined at 0.05 level. Different letters (a–f) on the graphs indicate significant differences between the treatments.

## Results

### Identification of the bacterium

**Fig. 1:**
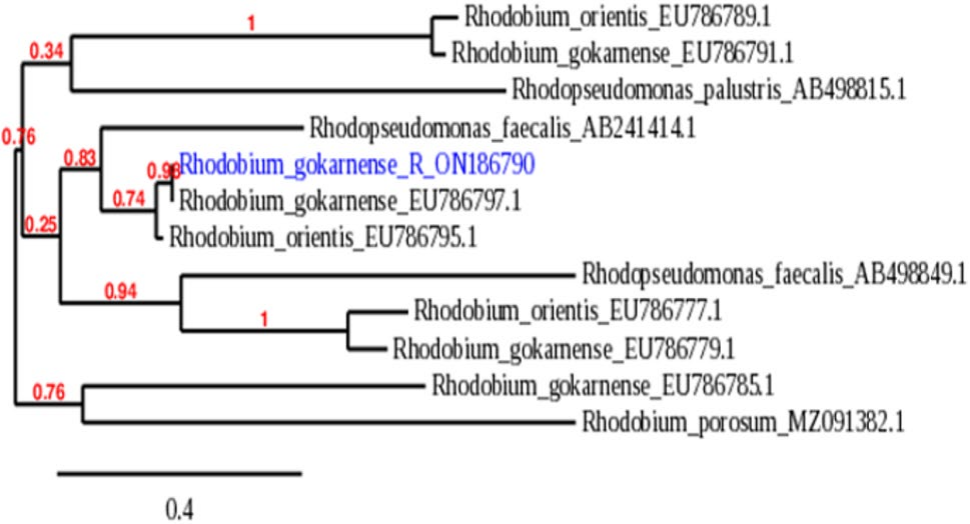
Phylogenetic tree based on genetic relationship of the case-studied purple non-sulfur bacterium *Rhodobium gokarnense* (R)

Morphological and biochemical characteristics of bacterial colonies are presented in Table 1 and the phylogenetic affiliation of the genetic material is presented in Fig. 1. According to the molecular analysis, the phylogenetic tree indicated that the strain exhibited 99% nucleotide base homology with the data in gene bank designating the strain as the PNSB *R. gokarnense*, the accession number of the herein studied strain is ON186790.

**Table 1:** Morphological and biochemical characterization of the case study purple non-sulfur bacterium *Rhodobium gokarnense*

Test	*Rhodobim jokarnense* (R)
Morphology	Rod
Cell size (µm)	0.4–0.6 × 1–1.8
Culture color	Pink to red
Motility	+
Carotenoids	+
Growth temperature range	25–35 °C
Optimum pH range	5–9
Nitrate reduction	−
Oxidase	−
Catalase	+
Hydrolysis of gelatine	−
Assimilation of	
Acetate	+
Lactate	−
Succinate	+
Malate	+
Citrate	−
Fructose	−
Glucose	+
Galactose	−
Lactose	−
Mannitol	+

### Growth

*Rhodobium gokarnense* was combined with the green alga *C. chodatii* to set up consortia containing successively increasing cell number (1*n*+, 1*n*−, 2*n*+, 2*n*−, 3*n*−, 3*n−*, 4*n*+, 4*n*−), in addition to the unialgal (C+/−) and unibcterial (R+/−) cultures; “*n*” refers to the cell number of 0.085 × 10^5^ algal cells/ml mixed with 1.8 × 10^5^ bacterial cells/ml giving a final O.D. of about 0.25 A at zero time of cultivation. Cultures and cocultures were followed for their growth, hydrogen evolution capacities and the cellular metabolism affecting this capacity. Growth (O.D.750 nm and cell number), light-induced oxygen evolution (photosynthesis), oxygen uptake in the dark (respiration). Cellular contents of chlorophyll, carotenoids, ascorbic acid and starch in addition to cellular exudations (reducing sugars, amino acids, soluble proteins) into the growth media were assessed.

**Fig. 2:**
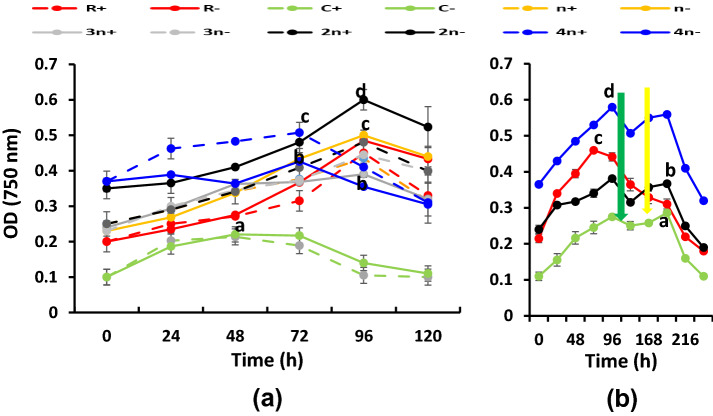
Optical density (O.D. 750 nm) of variously combined consortia of the purple non-sulfur bacterium *Rhodobium gokarnense* and the green alga *Coccomyxa chodatii* grown at sulfur replete (+) or deprived (−) conditions; **a** supplemented with malate in dim light, S.c  with malate/acetate mixture in dim light, **b** with malate/acetate, bicarbonate addition (green arrow) and sulfur re-addition (yellow arrow) in high light; *n* indicates cell number of bacteria (1.8 × 10^5^ cells/ml) combined with algae (0.085 × 10^5^ cells/ml); algae/bacteria ratio is 1/20

Figure 2 shows growth as the increase in optical density (O.D.750 nm) of the unialgal culture (C), unibacterial culture (R), and the bacterial/ algal combinations. In cultures supplemented with malate as the sole carbon source (Fig. 2a), the optical density was increasing up to its highest values at 72–96 h old cultures, decreased thereafter. The highest O.D. was exhibited at 2*n−* culture**,** among all cultures or culture combinations before leveling off at 120 h. The unialgal culture of *C. chodatii*, either replete (C+) or deprived of sulfur (C−), displayed the lowest O.D. However, when acetate and malate were both supplemented to the consortia (Fig. 2S.c), the top optical density was exhibited at 4*n*+ followed by 4*n−* and R−. The addition of bicarbonate, followed by sulfur at higher light intensity of 70 µmol/m^2^/s was applied to selected cultures of R−, C−, 2*n−* and 4*n−* representative to the most pronounced treatments. Upon addition of bicarbonate (10 mM) followed by re-addition of sulfur, cultures recommenced growth and the optical density of cultures (except R−) was transiently enhanced for the next 24 h only, following each addition, i.e., at 96 h and 192 h, respectively, before leveling off again at 216 and 240 h., respectively (Fig. 2b).

**Fig. 3:**
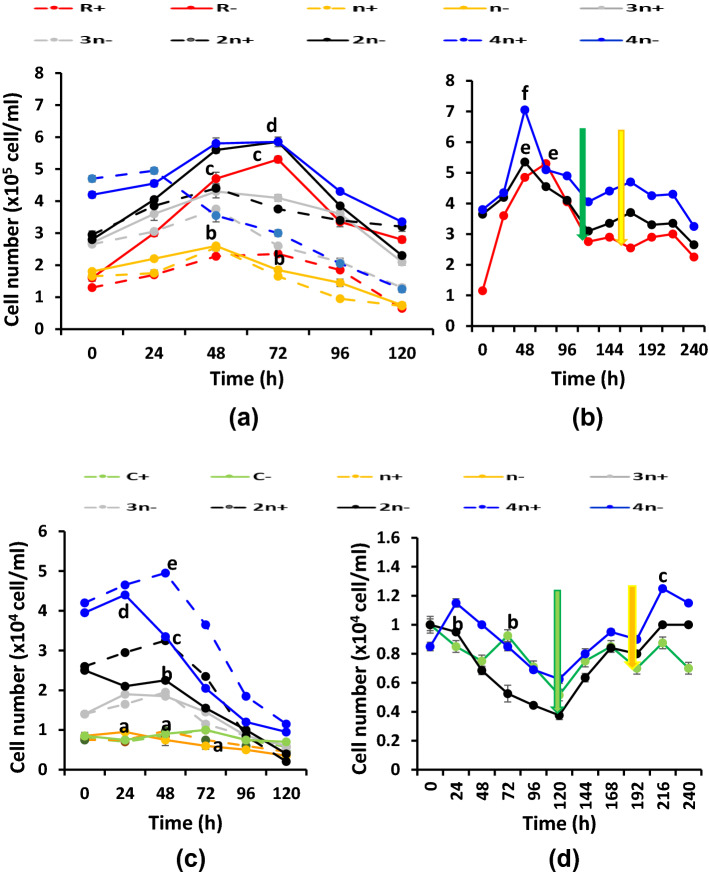
Cell number (Cell/ml) of variously combined consortia of the purple non-sulfur bacterium *Rhodobium* *gokarnense* (**a**, 3S e, **b**) and the green alga *Coccomyxa chodatii* (**c**, 3Sf, **d**) grown at sulfur replete (+) or deprived (−) conditions; supplemented with malate (**a**, **c**), with malate/acetate mixture (3**Se**, 3Sf), and bicarbonate (green arrow) and sulfur re-addition (yellow arrow) (**b**, **d**)

Cell numbers of bacteria and algae exhibited their highest values mostly at 48–72 h old cultures, at sulfur deprived or replete combinations; thereafter, cell number was mostly decreasing with the lapse of time as shown in Fig. 3a–f . Relying on malate only, bacterial cells exhibited their highest numbers at 4*n−* and 2*n−* consortia, higher than the unibacterial cultures indicating synergism with algae (Fig. 3a). However, algal cells displayed the highest values at 4*n+/−*; 4*n−*, which is promptly decreased just after 24 h (Fig. 3c). Acetic acid mostly did not enhance bacterial (Fig. 3S.e) nor algal (Fig. 3S.f) cell multiplication relative to malic acid but enhanced decline in bacteria. Bacterial cells did not respond to the addition of bicarbonate, sulfur or high light intensity (Fig. 3b) while growth of algae (Fig. 3d) resumed its increase after each addition.

**Fig. 4:**
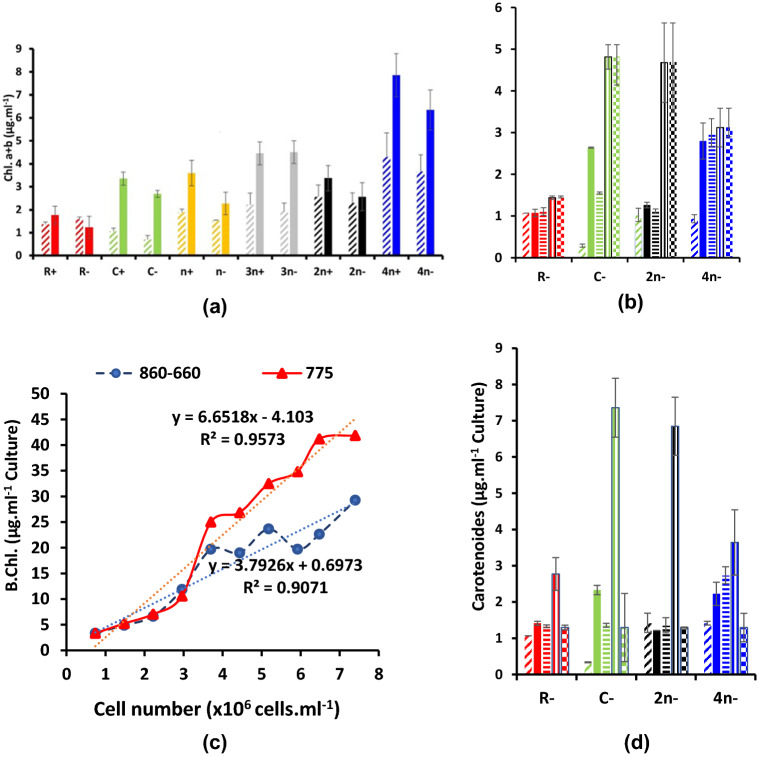
Pigment contents of variously combined consortia of the purple non-sulfur bacterium *Rhodobium* *gokarnense* and the green alga *Coccomyxa chodatii* grown at sulfur replete (+) or deprived (−) conditions; chlorophyll (a + b) contents in cultures supplemented with malate (**a**), with malate/acetate mixture (4Se), and bicarbonate and sulfur re-addition (**b**), bacteriochlorophyll (**c**) and carotenoids (4Sf, 4Sg, **d**); Patterns  refer to 0-time, 3rd-, 7th-, 7th day with HCO_3_^−^, and 7th day with HCO_3_^−^ and sulfur re-addition; respectively

Chlorophylls (a, b) were assessed daily; to conserve space and get a clear conclusion, only the 0-time and the third (last) day for each experiment are shown in Fig. 4. At malate (Fig. 4a), chlorophylls increased at all consortia and in the unialgal *Coccomyxa* cultures; 4*n*+ exhibited the highest chlorophyll contents among all cultures, due to the highest cell number inoculum. Sulfur deprivation decreased chlorophyll contents in all combinations,  except 3*n−* was slightly and 2*n−* was remarkably enhanced relative to their respective sulfur replete cultures. Acetate with malate induced similar chlorophyll increments with time but with lower magnitude of increase (Fig.4 S.e). Bicarbonate addition induced a sharp increase in chlorophyll contents particularly at C− and 2*n−* while sulfur re-addition did not (Fig. 4b). The relationship between bacteriochlorophyll assessed by absorbance of intact cells at 775, 860 and 660 nm and the cell number is presented in Fig. 4c. It shows a quantity of 4–5 µg/10^6^ cells of the PNS bacterium *R. gokarnense.* The absorbance of intact cells at 775 nm, multiplied by molecular weight showed more linearity with cell number (*R*^2^ = 0.957) than the difference between 860 and 660 nm (*R*^2^ = 0.907). Carotenoid contents at malate, malate/acetate and bicarbonate/sulfur addition were determined daily and shown in Fig. 4Sf, 4Sg. In malate only, *R. gokarnense* exhibited the highest contents (Fig. 4Sf) that is approached in malate/acetate only in consortia of 2*n−* and 4n +/−  (Fig. 4Sg). Bicarbonate addition extremely enhanced carotenoid contents while sulfur entirely abolished such enhancement (Fig. 4d).

**Fig. 5:**
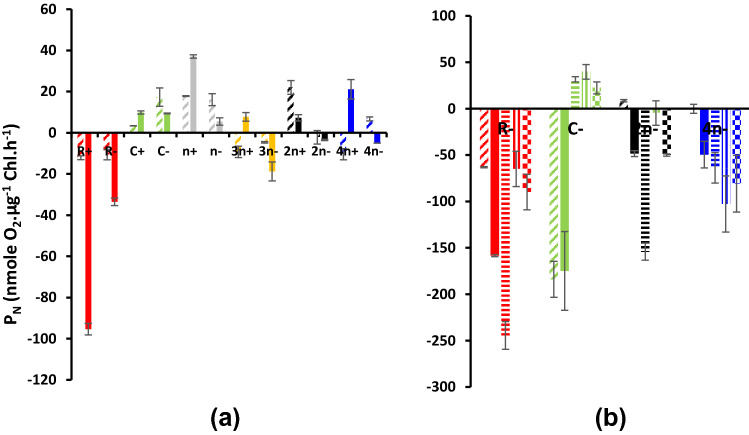
Net photosynthetic oxygen evolution (*P*_N_) as nmol O_2_ µg^−1^ Chl/h of consortia of the purple non-sulfur bacterium *Rhodobium gokarnense* and the green alga *Coccomyxa chodatii* grown at sulfur replete (+) or deprived (−) conditions; supplemented with malate (**a**), with malate/acetate mixture (5Sc), and bicarbonate and sulfur re-addition (**b**); the same patterns in Fig. 4 are identically applicable in this figure

**Fig. 6:**
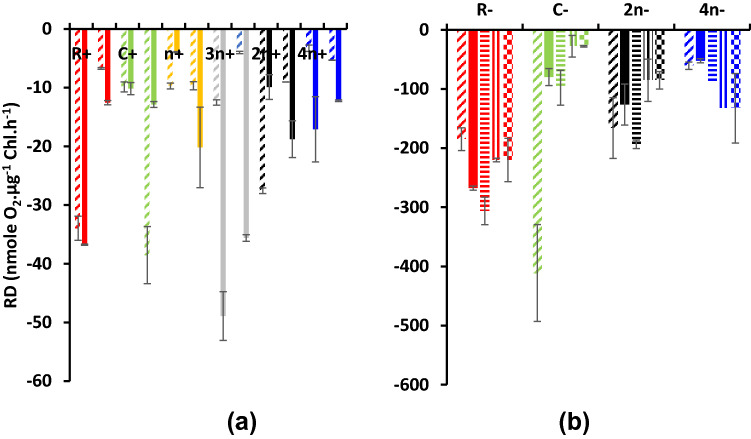
Dark respiratory oxygen uptake (*R*_D_) as nmol O_2_ µg^−1^ Chl/h of consortia of the purple non-sulfur bacterium *Rhodobium* *gokarnense* and the green alga *Coccomyxa chodatii* grown at sulfur replete (+) or deprived (−) conditions; supplemented with malate (**a**), with malate/acetate mixture (6Sc), and bicarbonate and sulfur re-addition (**b**); the same patterns in Fig. 4 are identically applicable in this figure

The net photosynthetic oxygen evolution (*P*_N_) is shown in Fig. 5. In malate supplemented cultures at dim light, PN was detectable in most cultures but in very low values with negative values in culture combinations of 3*n−*, 2*n−* and 4*n−* consortia Fig. 5a. Addition of acetate (Fig. 5Sc) absolutely inhibited net oxygen evolution in all cultures with high negative values recorded at the third day in *n*−, 2*n*− and 4*n*− consortia. Negative oxygen values means that respiration of both the bacterium and the alga consumed all the photosynthetically evolved oxygen in these cultures or culture combinations. Under the higher light intensity of 70 µmol/m^2^/s, the addition of bicarbonate and sulfur induced positive oxygen values at unialgal cultures of *Coccomyxa* (C−) whereas consortia remained of negative net photosynthesis (Fig. 5b). Respiratory oxygen uptake was enhanced by time, 3rd day vs 1st day in most sulfur deprived consortia supplemented with malate relative to sulfur replete, the highest at 3*n* followed by R− and 2*n*− (Fig. 6a). Respiratory oxygen uptake was higher in all cultures supplemented with malate/acetate than in malate alone (Fig. 6Sc) The addition of bicarbonate and sulfur did not accelerate respiration rates of unibacterial (R), unialgal (C), 2*n*− consortium but enhanced only at 4*n*− (Fig. 6b).

**Fig. 7:**
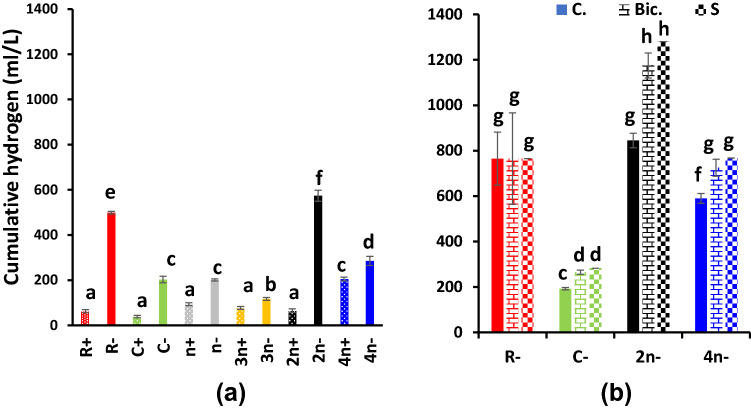
Cumulative hydrogen evolution as ml/l culture of consortia of the purple non-sulfur bacterium *Rhodobium gokarnense* and the green alga *Coccomyxa chodatii* at sulfur replete (+) or deprived (−) conditions; **a** supplemented with malate, (7Sc) with malate/acetate mixture, and **b** bicarbonate (yellow arrow) and sulfur re-addition (green arrow)

**Fig. 8:**
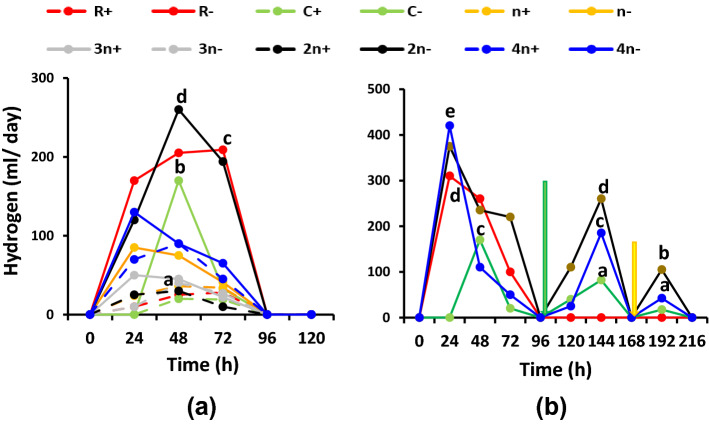
Kinetics of hydrogen evolution as ml H_2_/day per culture of consortia of the purple non-sulfur bacterium *Rhodobium* *gokarnense* and the green alga *Coccomyxa chodatii* at sulfur replete (+) or deprived (−) conditions; **a** supplemented with malate, (8Sc) with malate/acetate mixture, and **b** bicarbonate (yellow arrow) and sulfur re-addition (green arrow)

**Fig. 9:**
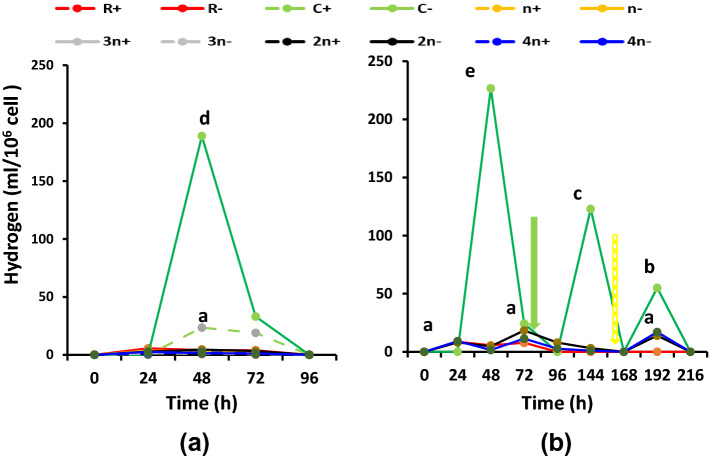
Specific hydrogen evolution as ml H_2_ 10^6^cells/day of consortia of the purple non-sulfur bacterium *Rhodobium* *gokarnense* and the green alga *Coccomyxa chodatii* at sulfur replete (+) or deprived (−) conditions; **a** supplemented with malate (9Sc) with malate/acetate mixture, and **b** bicarbonate (yellow arrow) and sulfur re-addition (green arrow)

Hydrogen evolution of the variously combined and treated cultures is presented in Fig. 7 for the cumulative values, Fig. 8 for the kinetics of evolution and Fig. 9 for the specific activity of algal and bacterial cells. Figure 7a shows the cumulative hydrogen evolution in malate-supplemented cultures at dim light. At such conditions, all sulfur deprived cultures were of higher hydrogen evolving capacity than the sulfur replete counterparts; the biggest volume of evolved hydrogen was recorded at the 2*n*− culture, followed by R− culture. The 4*n*− cultures, although containing double cell number, evolved only 50% hydrogen compared with the 2*n*−; the opposite occurred in sulfur replete cultures, i.e., 4*n*+ > 2*n*+. When acetic acid was supplemented in a mixture with malic acid to the cultures, hydrogen evolution exhibited almost an identical trend to that of malate only but enhanced to higher volumes (Fig. 7Sc). Also, its period of evolution was extended to 144 h instead of 72 h in the case of malate alone. The culture consortium 2*n*− remained the highest hydrogen evolving combination among all consortia, followed by R−. In malate/acetate fed cultures, 2*n*− evolved 830 ml H_2_/culture that is considerably higher than malate only (574 ml H_2_/culture), which indicates that acetate/malate synergistically enhanced hydrogen evolution more efficiently in consortia than in uni-cultures. Figure 7b shows hydrogen evolution in sulfur deprived unibacterial—(R−), unialgal (C−) cultures as well as in 2*n*− and 4*n*− cultures but at a higher light intensity of 70 µmol/m^2^/s. At such conditions, bicarbonate supplementation induced recommencement of hydrogen evolution at all cultures to higher cumulative levels. Sulfur readdition further enhanced hydrogen evolution. Also, the consortium 2*n*−, as usual, exhibited the highest activity, followed by 4*n*− while the unibacterial culture R− was also enhanced by higher light. However, the unibacterial cultures did not respond to bicarbonate supplementation nor to sulfur readdition (Fig. 7b).

Figure 8 displays the kinetics of hydrogen evlution by uni-cultures and consortia. In most cultures, the major part of hydrogen was evolved within the first 24 h, continued to evolve at minor rates for the next two days, i.e. total of 72 h but at a sharply decreasing attitude; this applies to malate (Fig. 8a) or malate/acetate fed cultures (Fig. 8Sc) at dim light. While the unibacterial PNSB culture *R. gokarnense* (R−) evolved its major portion of hydrogen at day 1; continued for two more days but at considerably lower rates, the unialgal culture *C. chodatii* (C−), as always, evolved all its hydrogen at day 2 only, in -malate or malate/acetate-fed cultures (Fig. 8S). Hydrogen evolution usually ceases at 48–72 h old cells in most cultures. Howevet, at the higher light intensity, addition of bicarbonate and sulfur recpmmenced hydrogen evolution for more or two more days, depending on the type of culture or consortium (Fig. 8b). The unialgal culture, remained strict to evolve hydrogen over the second 24 h only even at additional bicarbonate and sulfur. Another form of hydrogen kinetics is presented in Fig. 8′Sa–c in the supplementary file.

Specific hydrogen evolution as ml H_2_ 10^6^ cell/day revealed that the algal cells of *C. chodatii* are much more efficient in hydrogen evolution capacity than per cell of the purple non-sulfur *R. gokarnense* either in malate (Fig. 9a), malate/acetate (Fig. 9Sc) or when bicarbonate or sulfur were provided (Fig. 9b).

Ascorbic acid in the bacterial strain *R. gokarnense* exhibited generally higher content than the alga *C. chodatii* (Table 2). The addition of acetic acid mostly reduced cellular contents of ascorbic acid indicating less oxidative stress relative to malic acid alone. No characteristic attitude of ascorbic acid among the various combinations or due to sulfur deprivation.

**Table 2:** Ascorbate and starch contents in cells of the variously-treated culture consortia (µg/ml culture)

Treatments	Ascorbate	Starch
Malic acid		
R+	3.49 ± 0.49	0.27 ± 0.01
R−	11.96 ± 5.76	0.16 ± 0.01
C+	2.16 ± 0.16	0.17 ± 0.02
C−	9.01 ± 4.96	0.19 ± 0.02
*n*+	3.9 ± 1.47	0.18 ± 0.01
*n*−	3.22 ± 2.15	0.17 ± 0.01
2*n*+	5.25 ± 0.12	0.17 ± 0.01
2*n*−	5.11 ± 0.80	0.22 ± 0.06
4*n*+	7.80 ± 1.36	0.16 ± 0.01
4*n*−	8.87 ± 2.40	0.18 ± 0.01
Acetic acid		
R−	5.28 ± 0.61	0.28 ± 0.09
C+	1.68 ± 0.06	0.22 ± 0.03
C−	2.41 ± 0.61	0.22 ± 0.03
*n*+	3.10 ± 0.81	0.27 ± 0.08
*n*−	2.41 ± 0.01	0.30 ± 0.05
2*n*+	6.32 ± 0.58	0.20 ± 0.04
2*n*−	2.07 ± 0.12	0.21 ± 0.02
4*n*+	3.26 ± 0.81	0.26 ± 0.07
4*n*−	4.25 ± 0.50	0.20 ± 0.07
Bicarbonate and sulfur		
R−	14.284	0.172
R− bic	18.865	0.141
(R−) + bic + S	26.142	0.162
C−	2.41	0.220
C− bic	1.078	0.182
(C−) + bic + S	1.617	0.245
2*n*−	19.135	0.185
2*n*− bic	3.773	0.171
(2*n*−) + bic + S	5.390	0.173
4*n*−	4.25	0.168
4*n*− bic	1.426	0.170
(4*n*−) + bic + S	2.426	0.179

Starch content was higher in acetic/malic supplemented cultures relative to malic acid alone (Table 2), without obvious difference among the different combinations or sulfur national status, replete or deprived.

Exudates of reducing sugars, amino acids and soluble proteins from the bacterium *R. gokarnense*, the alga *C. chodatii* and their consortia were detected and presented in Table 3. Reducing sugars were almost of similar values at the various cultures or combinations. Amino acids exhibited their lowest values at unialgal cultures and their highest values in unibacterial cultures while the consortium 2*n*− was in between but closer to the unibacterial contents. Sulfur deprivation slightly enhanced amino acids leakage in R− and C− compared with sulfur replete R+ and C+ but the opposite was found in 2*n*−. Soluble proteins exhibited a trend similar to that of amino acids.

**Table 3:** Reducing sugars, amino acids and soluble protein exudates into the culture media) as µg/ml culture

	Reducing sugars	Amino acids	Soluble proteins
Aerobic			
R+	0.09 ± 0.01	36.25 ± 0.72	6.00 ± 1.06
R−	0.09 ± 0.01	1 ± 0.29	24.33 ± 2.84
C+	0.09 ± 0.01	4 ± 1.15	8.00 ± 0.86
C−	0.09 ± 0.01	9 ± 2.46	4.17 ± 0.60
2*n*+	0.10 ± 0.01	9 ± 2.46	13.67 ± 3.16
2*n*−	0.09 ± 0.01	4 ± 0.29	17.50 ± 3.96
Anaerobic			
R+	0.10 ± 0.01	27.8 ± 4.87	19.83 ± 2.24
R−	0.11 ± 0.01	30.16 ± 4.40	19.00 ± 1.32
C+	0.09 ± 0.01	3.16 ± 0.33	1.83 ± 0.32
C−	0.08 ± 0.01	4.66 ± 1.94	3.17 ± 1.0
2*n*+	0.09 ± 0.01	22.33 ± 3.58	15.00 ± 1.60
2*n*−	0.08 ± 0.01	20.5 ± 3.62	10.66 ± 0.71

## Discussion

The capacity of photosynthetic or fermentative hydrogen evolution by microorganisms (algae, cyanobacteria and bacteria) is, certainly constrained by various metabolic barriers; above which, oxygen is a powerful inhibitor of the hydrogen evolving enzymes (hydrogenase and nitrogenase). Accordingly, it is difficult to approach the theoretical hydrogen:oxygen ratio of 2:1 via PSII-photolysis of water or in sugars during fermentation. In this work, a number of manipulations tuning cultures’ oxygen content were applied seeking a breakthrough in enhancing the magnitude of hydrogen evolution. Namely, various consortia of the newly isolated PNSB strain *R. gokarnense* ON186790 “R” and the green alga *C. chodatii* “C” were set up; both microorganisms are explored for the first time in biohydrogen production field. These consortia are studied at sulfur deprivation/re-addition, malate and malate/acetate supplementation, dim/high light intensities and bicarbonate addition. Consortia grow compatibly in inorganic medium enriched with malic acid, but more with malic/acetic acids. They evolved hydrogen amounts more than their separate cultures**,** indicating the mutual synergism in supporting growth and hydrogen evolution of each other (*R. gokarnense* on *C. chodatii* and vice versa, i.e., algae on bacteria). In particular, the consortium 2*n*− (*n* = 1.9 × 10^5^ cell/ml, 1:20 (*Coccomyxa*:*Rhodobium*), sulfur deprived) demonstrated its perfection with the planned aim of this work, i.e., the highest possible cumulative hydrogen evolution level more than their respective monocultures or other consortia. Such consortium is characterized by negative net photosynthesis in the light, i.e., net photosynthetic oxygen evolution was inhibited and turned oxygen uptake in the light, by respiration of both organisms. Algae and bacteria at this combination (2*n*−) may be at their ideal proportions to economize oxygen evolution for hypoxia with electron transport in favor of hydrogen evolution, as none of the other consortia was competent with this consortium regarding the hydrogen evolution values. The consortium 4*n−* resulted in less hydrogen evolution, may be due to the positive oxygen content, which started to accumulate in this consortium by the more algal cells than in 2*n−*. Such findings at 2*n−* and 4*n−* are indicative for synergized oxygen tuning and hydrogen evolution metabolism, same as in growth. In accordance with this, Ban et al. (2018) reported that in co-cultures of *Pseudomonas* sp. strain D and *C. reinhardtii*, the relative O_2_ content in the headspace plus the dissolved oxygen in the culture medium were rapidly consumed by bacterial growth, resulting in a completely anaerobic environment. These findings clue that a balanced cell number of bacteria and algae is needed for sustainable hydrogen evolution by consortia.

Kinetics of hydrogen evolution displayed that the consortia 4*n−* and 2*n−* as well as the bacterial culture R− evolved their major portion of hydrogen on the first 24 h, continued to evolve hydrogen for the next two days, i.e. 72 h but at sharply decreasing rates before ceasing. However, the sulfur deprived green alga *Coccomyxa* (C−) did not evove any hydrogen at the first day under any circumstance of nutrition (malate or malate/acetate), in single culture or combination; it evolved all its hydrogen over the second 24 h, i.e., at day 2 only, with a lag of the first 24 h before hydrogen becomes detectable. However, addition of bicarbonate followed by sulfur at the high light intensity recommenced hydrogen evolution for one or two more days, depending on the type of consortium, i.e. the proportion of algae to bacteria, which might lead to inhibitory concentrations of oxygen.

The specificity of hydrogen evolution as ml H_2_/ 106 cell/ day, of *C. chodatii*, is characterized by three phenomena: (a) much higher hydrogen evolution per cell of *C. chodatii* than per cell of *R*. *gokarnense* either in malate, malate/acetate or when bicarbonate or sulfur were provided, dim or high light intensity (b) hydrogen evolution for only 24 h and (c) a lag of 24 h before hydrogen becomes detectable. However, the higher productivity, i.e., higher cumulative hydrogen per culture resulted from bacteria (80–90%) due to higher cell density (20 times) than that of the alga, durability period, which is 2–3 days in bacteria whereas only one day in algae, in addition to an inferred integrative metabolism. If the algal cells are increased, photosynthesis concomitantly increased and oxygen tension might become inhibitory; this happened at 4*n−*. The share of *R.* *gokarnense* equals 2.5 times hydrogen that of the unialgal *C. chodatii* cultures in malate fed cultures, increased to 4.5 times with acetate. Specific hydrogen increased with light intensity (Kim et al. 2006a, b; Park and Moon 2007; Markov et al. 2006; Vijayaraghavan et al. 2010).

Starch contents of consortia were higher at acetic/malic supplementation relative to malic acid alone, without obvious differences among the different consortia or sulfur nutritional status, replete or deprived. Starch accumulation/degradation is a common feature of sulfur deprivation since discovered in *C. reinhardtii* by Melis et al. (2000). Also, co-culturing bacteria such as *Bradyrhizobium japonicum* (Xu et al. 2016), *Azotobacter chroococcum* (Xu et al. 2017), *Pseudomonas* sp. (Ban et al. 2018) and *Thuomonas intermedia* (He et al. 2018) favors high starch accumulation in *Chlamydomonas*. The fermentative bacteria such as *Lactobacillus amylovorus*, *Vibrio fluvialis* and *Clostridium butyricum* can degrade the *Chlamydomonas* biomass and excrete organic acids such as ethanol, formate, acetate, propionate and butyrate, which can be used by the partner-in consortia PNSB *Rhodobacter sphaeroides*, *Rhodobacter* *capsulata, Rhodospirillum rubrum* and *Rhodobium marinum* (Kawaguchi et al. 2001; Ike et al. 1997), *Rhodobacter sphaeroides* (Kim et al. 2006a, b) to photoproduce H_2_ via photo-fermentation. Starch-enriched *Chlamydomonas* biomass can be also used directly by some heterotrophic bacteria to produce H_2_ or in collaboration with photosynthetic PNS bacteria (Fakhimi et al. 2020). Respiratory or fermentative metabolism of sugars, derived from starch via glycolysis, supplies a significant number of electrons to the PQ-pool via the plastidial type II NAD(P)H dehydrogenase (NDA2) complex and thereby for the expressed hydrogenase, i.e. PSII-independent hydrogen evolution (Mignolet et al. 2012; Volgusheva et al. 2013; Hong et al. 2016). However, when linear electron transport is limited, neither efficient starch degradation nor high hydrogenase activity would result in strong H_2_ production (Nagy et al. 2016). Starch metabolism, besides being a source of electrons, accelerates oxygen uptake and thus hypoxia installation. Also, Assimilation of acetic and malic acid also participate in oxygen consumption and connected to H_2_ production, similar to former findings (Jurado-Oller et al. 2015; Gibbs et al. 1986; Bamberger et al. 1982; Fakhimi et al. 2020). Acetic acid probably being the metabolite linking dark H_2_ production with H_2_ photoproduction (Fakhimi et al. 2019, 2020), although the theoretical values of acetic acid-based yield are not usually reached due to different limitations as the use of photosynthetic bacteria in such integrative systems often requires two-stage bioreactors due to the growth incompatibility (Hallenbeck and Liu 2016). However, acetate uptake is greatly dependent on oxygen availability; low levels of oxygen allow for low acetate uptake rates, but paradoxically, lead to efficient and sustained production of hydrogen (Jurado-Oller et al. 2015). Organic exudates may be mutually excreted and exchanged for growth and hydrogen evolution. In this respect, Fakhimi et al. (2020) depicted some metabolites that can be potentially exchanged between algae and other microorganisms during growth and H_2_ production conditions. In this work, reducing sugars, amino acids and soluble proteins were detected in consortia of *C. chodatii* and *R. gokarnense*. Secreted end products can be theoretically used by bacteria as electron donors for H_2_ production, and some of them have been probed at an empirical level using *Chlamydomonas*–PNSB bacteria cultures (Miyamoto et al. 1987; Miura et al. 1992).

### Light, bicarbonate and oxygen

Light, bicarbonate and sulfur are prime factors orchestrate the highly complicated process of photosynthetic oxygen evolution from PSII activity by different mechanisms. Direct photosynthetic (PSII → PSI → FDX1 → HYDA1), indirect photosynthetic (PQ → PSI → FDX1 → HYDA1) and photofermentative (P(OA)FR → PSI → FDX1 →  → HYDA1) are hydrogen evolution pathways described in *C. reinhardtii* by González-Ballester et al. (2017); PFR is Pyruvate Ferredoxin Reductase, HYDA1 is the primary hydrogenase, OA is oxaloacetate, FDX1 is ferredoxin 1. In sulfur deprived cells, the electrons feeding H_2_ production originate mostly from the remaining PSII activity (Chochois et al. 2009), which decreased by only 25% (Volgusheva et al. (2013), concomitant with 25% decrease in cellular sulfur content **(**Nagy et al. 2018a). The electron transport from PSII was completely blocked during the anaerobic phase preceding H_2_ formation; hydrogenase partly removes this block, thereby permitting electron flow from water oxidation to hydrogen **(**Nagy et al. 2018a). Meanwhile, sulfur reserves such as sulfolipid, mostly in chloroplast membrane in the form of sulfoquinovosyl diacylglycerol (SQDG), mobilize upon sulfur deprivation, as a major internal sulfur source for protein synthesis at the early phase of sulfur starvation in *Chlamydomonas reinhardti* (Sugimoto et al., 2007, 2010). Based on these observations, therefore, it is very likely that the quantity of sulfur per photosynthetic reaction center decreases only moderately and may not substantially hinder PSII structure and activity. In this respect, Abdel-Basset and Bader (1998) reported that the PSII-blocker DCMU was inhibitory only at early and transition state of installing anaerobiosis. In this work, net photosynthetic oxygen evolution was only detectable in few cultures fed with malate, absolutely undetectable upon addition of acetic acid; otherwise, it was negative, i.e., respiration of consortia consumed all oxygen and established anoxic conditions necessary for hydrogen evolution. In this work, the major part of the experiments was conducted at dim light of only 2 µmol/m^2^/s. Dim light was applied to secure a continuous flow of electrons for proton reduction and simultaneously avoid high light intensity drawbacks, substantially the inhibitory effect of enhanced photosynthetic oxygen evolution on the hydrogen evolving enzymes (nitrogenase and hydrogenase). Drawbacks include, in addition, photooxidation and overexcitation of the redox components, oxidative stress and singlet oxygen species, and down regulation of PSII of the green alga *C. chodatii* as well as in its consortia with the PNSB *R. gokarnense*. In this respect, low oxygen levels at low light intensities in acetate‑containing media were found to elicit and improve photohydrogen production in mixotrophic non-stressed nutrient-replete *Chlamydomonas* cultures **(**Jurado-Oller et al. 2015), in a slow, continued, but sustained level of H_2_ production. Tsygankov et al. (2006) demonstrated possible sustained H_2_ photoproduction by a sulfur-deprived *C. reinhardtii*, under strictly photoautotrophic conditions without acetate or any other organic substrate; pre-grown with 2% CO_2_ under low light conditions along with a special light regime. Applying higher light intensity of 70 µmol/m^2^/s to the studied consortia of *C. chodatii* and *R. gokarnense* recommenced hydrogen evolution but transiently for one or two more days before ceasing again, and to a less level than the original first stage at dim light of 2 µmol/m^2^/s, most probably due to oxygen accumulation. In the literature, Various hydrogen evolution maxima were recorded in the literature in relation to light intensity, alternating dark/light, intermittent light or light spectra even with the same *C. reinhardtii* (Laurinavichene et al. 2004). Using ‘‘gain-of-function’’ mutations, Forster et al. (2005) isolated at least four such very high light resistant (*VHL*^*R*^) mutations in *C. reinhardtii*, that permit near maximal growth rates at light intensities lethal to wild type.

The addition of bicarbonate, also, induced growth recommencement of *C. chodatii* and *R. gokarnense*, but transiently over another one more day, although it did not attain the original levels of the first 48 h before finally ceasing again. Bicarbonate or sulfur addition was accompanied with positive oxygen values, i.e., the cultures become autotrophic and the oxic environment started to establish, i.e., a transient autotrophic hydrogen evolution occurred before inhibition by photosynthetic oxygen. Inorganic carbon (CO_2_ or bicarbonate) plays three roles by three different mechanisms in oxygenic photosynthesis: CO_2_ fixation into carbohydrates (Calvin cycle), CCM and Warburg’s effect (the role of bicarbonate in maintaining PSII activity). Hong et al. (2016) recorded that during H_2_ production phase of S deprivation, the concentrations of starch and H_2_ in CCM-induced cells were remarkably enhanced by 65.0% and 218.9% compared to that of CCM-uninduced cells, respectively. Warburg’s effect is a unique stimulatory role of CO_2_ in the Hill reaction (i.e., O_2_ evolution) has been studied by Govindjee and his coworkers (reviewed in Shevela et al. 2012). Wydrzynski and Govindjee (1975) discovered a definite bicarbonate effect on the electron acceptor (the plastoquinone) side of PSII. Nagy et al. (2018b), by applying a simple catalyst (glucose and glucose oxidase) to remove the evolved O_2,_ enabled cultures to remain photosynthetically active for several days, keeping the Calvin–Benson cycle inactive by substrate limitation and preserving hydrogenase activity, with the electrons feeding the hydrogenases mostly derived from water. The amount of H_2_ produced is higher as compared to the sulfur-deprivation procedure and the process is photoautotrophic, ascorbic acid in *R. gokarnense* exhibited generally higher content than *C. chodatii*. No characteristic attitude of ascorbic acid content among the various consortia or due to sulfur deprivation. The addition of acetic acid, mostly reduced cellular contents of ascorbic acid indicating less oxidative stress or more relief of PSII, relative to malic acid alone. Simultaneous with reduced ascorbic acid, the cumulative hydrogen evolution, significantly enhanced. The imposed oxidative stress early on sulfur deprivation, most probably involving the formation of singlet oxygen (^1^O_2_) in PSII, which leads to an increase in the expression of GDP-l-galactose phosphorylase, exerting oxidative stress playing an essential role in ascorbate biosynthesis (Nagy et al, 2016, 2018a). In the absence of a functional donor side and sufficient electron transport, PSII reaction centers are inactivated and degraded. When accumulated to the mM concentration range, ascorbate may contribute to the inactivation of the Mn-cluster in the OECs (oxygen evolving complexes) due to its reducing capacities and may provide electrons to PSII, albeit at a low rate. The inactivation of PSII is, therefore, a complex and multistep process, which may serve to mitigate the damaging effects of sulfur limitation (Nagy et al. 2018a). Finally, At the end of the H_2_-producing period, sulfur limitation may restrain cell growth and viability; the entire cell system starts to degrade, and genes related to apoptosis and protein degradation are upregulated (Toepel et al. 2013).

## Conclusions

- Bacterial or algal cells were of higher numbers in consortia than in their uni-cultures, indicating no inhibitory, antagonistic or allelopathic but rather the supportive synergistic effect of algae on bacterial growth and vice versa, i.e., bacteria on algae.
  The consortium revealed full perfection for the highest hydrogen evolution level is 2*n−* (cell density of 1.9 × 10^5^ cell/ml sulfur deprived of the Chlorophycean *Cocomyxa chodatii* and the PNSB *R. gokarnense* at a ratio of 1:20).

- Dim light seemed economically feasible alternative to high light, as the values of hydrogen are comparable or even more and the costs of artificial illumination can thus be eliminated.
  All hydrogen evolved in this work is photofermentative; malate but more malate/acetate mixtures enhanced hydrogen evolution of algal and bacterial consortia at dim light.
- Cells of the green alga *C. chodatii* evolved the highest hydrogen per cell compared with the bacterial cells but for only 24 h, with a one-day lag.
- Bicarbonate or sulfur addition resumed hydrogen evolution for additional 24–48 h, indicating elasticity of the process and viability of the cells.
- Yet, algal/bacterial (cyanobacterial) consortia are worth further exploration as their variables are many, e.g., different strains, cell number, proportions, light intensity and spectra, different organic acids, manipulating autotrophic vs heterotrophic life and nitrogenase/hydrogenase optimization, etc., which would improve hydrogen yield.

## Supplementary Information

Below is the link to the electronic supplementary material.

Supplementary file1 (DOCX 1129 kb)

## Data Availability

https://www.springernature.com/gp/authors/research-data-policy/data-availability-statements/12330880.
