# Supplementary material for: Tuning photosynthetic oxygen for hydrogen evolution in synergistically integrated, sulfur deprived consortia of Coccomyxa chodatii and Rhodobium gokarnense at dim and high light
Source: Photosynth Res. 2022 Nov 23;155(2):203–18. doi: 10.1007/s11120-022-00961-4 (PMC9879849; doi:10.1007/s11120-022-00961-4)
Supplement: Supplementary file 1 — Supplementary file1 (DOCX 1129 kb) [file 11120_2022_961_MOESM1_ESM.docx]

**Fig. 2Sc**

**Fig. 3Se**

**Fig. 3Sf**

**Fig. 4S e**

**Fig. 4Sf**

**Fig. 4Sg**

**Fig. 5Sc**

**Fig. 6Sc**

**Fig. 7Sc**

**Fig. 8Sc**

**Fig. 8’ Sa**

**Fig. 8’ Sb**

**Fig. 8’ Sc**

**Fig. 9Sc**
